# Psilocybin and MDMA reduce costly punishment in the Ultimatum Game

**DOI:** 10.1038/s41598-018-26656-2

**Published:** 2018-05-29

**Authors:** Anthony S. Gabay, Robin L. Carhart-Harris, Ndaba Mazibuko, Matthew J. Kempton, Paul D. Morrison, David J. Nutt, Mitul A. Mehta

**Affiliations:** 1 Department of Neuroimaging, Institute of Psychiatry, Psychology & Neuroscience, King’s College London, London, United Kingdom; 2 Department of Psychosis Studies, Institute of Psychiatry, Psychology & Neuroscience, King’s College London, London, United Kingdom; 3 Psychedelic Research Group. Neuropsychopharmacology Unit. Centre for Academic Psychiatry, Imperial College London, London, United Kingdom

## Abstract

Disruptions in social decision-making are becoming evident in many psychiatric conditions. These are studied using paradigms investigating the psychological mechanisms underlying interpersonal interactions, such as the Ultimatum Game (UG). Rejection behaviour in the UG represents altruistic punishment – the costly punishment of norm violators – but the mechanisms underlying it require clarification. To investigate the psychopharmacology of UG behaviour, we carried out two studies with healthy participants, employing serotonergic agonists: psilocybin (open-label, within-participant design, N = 19) and 3,4-methylenedioxymethamphetamine (MDMA; placebo-controlled, double-blind, crossover design, N = 20). We found that both MDMA and psilocybin reduced rejection of unfair offers (odds ratio: 0.57 and 0.42, respectively). The reduction in rejection rate following MDMA was associated with increased prosociality (R^2^ = 0.26, *p* = 0.025). In the MDMA study, we investigated third-party decision-making and proposer behaviour. MDMA did not reduce rejection in the third-party condition, but produced an increase in the amount offered to others (Cohen’s *d* = 0.82). We argue that these compounds altered participants’ conceptualisation of ‘social reward’, placing more emphasis on the direct relationship with interacting partners. With these compounds showing efficacy in drug-assisted psychotherapy, these studies are an important step in the further characterisation of their psychological effects.

## Introduction

Humans live in complex social environments and have evolved a number of social cognitive mechanisms which aid interaction with others^1,2^. Accumulating evidence suggests that alterations in social cognition exhibited across psychiatric disorders include interpersonal interactions, as measured by social decision-making paradigms^3,4^. Social decision-making tasks are used to investigate the mechanisms underlying high-level concepts such as trust, social norm enforcement and cooperation. It is clear that these pervade interactive behaviour. Investigating the psychopharmacology in healthy individuals will help to establish both the pharmacological and psychological mechanisms underlying such concepts, and may also inform on potential treatment targets in people for whom alterations of these disrupt their quality of life.

Psilocybin and 3,4-methylenedioxy-methamphetamine (MDMA) are two compounds which have recently shown promise in treating depression, trauma and anxiety^5–7^. How they affect different aspects of cognition needs further exploration. With MDMA-assisted psychotherapy for post-traumatic stress disorder recently approved for Phase III clinical trials, it is timely to expand upon the psychological profile of these potential treatments. Regarding social cognition, this work has already begun. Evidence suggests both psilocybin and MDMA can affect processes such as empathy, emotion recognition and responses to social exclusion^8–11^. With social decision-making tasks increasingly being used to assess how social interactions are altered in clinical populations^12,13^, it is appropriate to investigate how these may be altered following administration of these compounds.

We present two psychopharmacological studies investigating behaviour in the Ultimatum Game^14^. The UG has a rich literature exploring behaviour^15^ and neural correlates^16,17^ during the task, and has received the most research attention of the different social decision-making paradigms. The classic UG is a two-player game. One player, the proposer, is given a sum of money and asked to split it with the other player, the responder. The responder decides whether to accept or reject the offered split. If the offer is accepted, the money is divided as offered. If the offer is rejected, neither player receives any money at all.

While the ‘economically rational’ decision is for the responder to accept any amount offered in a one-off interaction, studies find that the majority of people reject low, unequal offers^15,17^. It has been proposed that rejection is prosocial, and is a form of altruistic punishment – punishment of social norm violations which are costly to the self^18,19^. While there is some debate around the mechanisms underlying this costly punishment – whether it be driven by purely ‘prosocial’ considerations or as an act of revenge – it is accepted to be a reaction to violations of social norms related to unfair treatment^20^.

Crockett *et al*. have carried out studies which implicate the serotonin (5-HT) system in social decision-making^21–23^. They report that reducing 5-HT availability through acute tryptophan depletion (ATD) increases rejection rates of moderately, but not very unfair offers (30% and 20% of the total stake, respectively)^21,23^. Increasing availability of 5-HT through single doses of selective serotonin reuptake inhibitors (SSRI) reduced rejection rates of moderately unfair offers^22^. These were interpreted as 5-HT influencing harm aversion, with offer rejection harming the other through denial of reward. These studies provide evidence that the UG is sensitive to serotonergic challenge. Therefore, one could expect psilocybin and MDMA, both having serotonergic agonist action, to also modulate UG behaviour.

5-HT appears to play a role in responder behaviour in the classic UG. However, its effect on third-party behaviour has received limited attention, thereby limiting the psychological interpretation of the changes seen. A series of studies have included a third-party condition^24–26^. Here the proposer makes an offer to a third party, and participants are asked to decide in the responder’s place. An accepted offer is divided between the proposer and the third party; if it is rejected, neither player receives any money. These studies found that participants reject low offers in this condition, suggesting that rejection is driven by equality considerations rather than reactions to *personal* unfair treatment.

Crockett *et al*.^21^ reported reduced willingness to pay a cost to punish in a third-party punishment game following ATD, but by their own acknowledgment the task was not directly comparable to the UG. Here, we report two studies to address these questions; one with psilocybin, and the other with MDMA. The latter included a third-party condition.

The two compounds have well-described subjective effects, but differentially affect the serotonergic system. Psilocybin has strong psychedelic effects, mediated by 5-HT2A receptor agonism^27^. Recent work has begun investigating its effects on social cognition^9,10^, but not on social decision-making. Therefore, we included the UG in a larger study on psilocybin in order to provide preliminary evidence here. MDMA produces potent emotional and social effects^28^. It elicits dopamine, noradrenaline and serotonin release^29^. Serotonin is thought to be primarily responsible for mediating its prosocial and euphoric effects^30,31^, due to a 10-fold higher affinity of MDMA for the 5-HT transporter than the catecholamine transporters^32^.

While the pharmacological profile of MDMA is broader than psilocybin, it is well-known for altering social and emotional cognition^8,11^. This study was not aimed at further defining the serotonergic mechanisms of the UG, but instead to improve our understanding of the role of this specific compound.

Given the evidence from the SSRI studies above, we hypothesised that both psilocybin and MDMA would reduce rejection rates of unfair offers in a first-person condition. In the MDMA third-party condition, we hypothesised that MDMA would not alter rejection rates; under the assumption that equality considerations would remain unchanged when not directly involved in the outcome. We included a non-social control condition in both studies, and hypothesised that neither compound would alter behaviour in this condition. Finally, we hypothesised an increase in the percentage offered when acting as proposer following MDMA administration.

## Study one – the effects of psilocybin

### Methods

The data presented in this section were collected as part of a larger study investigating the effects of a src-kinase inhibitor on the effects of psilocybin. This study involved three visits: a drug-free screening, and two further sessions, where the src-kinase inhibitor or placebo was administered (double-blind, randomised) and later in the day psilocybin (open-label) was given to all subjects. The current manuscript presents the UG data from the drug-free screening session and psilocybin-alone session. All other data from this study will be reported elsewhere.

#### Participants

Twenty-three male participants were recruited from the community, all of whom had previous experience of a psychedelic drug. All participants gave written informed consent and were financially compensated for their time. The study received ethical approval from King’s College London’s Psychiatry, Nursing and Midwifery Research Ethics Committee (PNM/14/15-11). All experiments were performed in accordance with relevant guidelines and regulations. See Supplementary Information S1 for the exclusion criteria for this study and details relating to participants excluded. 20 participants completed the study (mean age 26.6, SD 7.1, range 19–47).

#### Experimental procedure

Participants attended three sessions. The analyses presented here use data from the drug-free screening session and the psilocybin session; thus this represents an open-label designed analysis of psilocybin vs a drug-free session (time between drug-free session and psilocybin session: mean 36.0 days, SD 33.6, range 6–141).

For a full description of the experimental psilocybin day, see the Supplementary Information S2. Briefly, in the mid-afternoon 2 mg psilocybin was administered via intravenous infusion over 2 minutes during the neuroimaging session. The UG was performed 60 minutes later, when most of the acute effects had diminished, thus removing the confound of profound perceptual alterations during the task. There is evidence of enduring cognitive effects of acute psilocybin administration^33^.

#### The Ultimatum Game

This version of the UG had two ‘offer origin’ conditions: first-person and random computer-generated offer (random-generated).

In the first-person condition, participants decided to reject or accept an offer made by another person. Participants were told that the offers were collected as part of another study in the wider research project. They were led to believe that previous participants would be paid a proportion of the money earned, based on the responses of the participants in the current study. In the random-generated condition, the participant was told that the offer they received was a random, computer-generated offer. In this condition, their decision solely affected their own payoff.

Participants were told that they would be paid one percent of the total amount earned during the course of the study; in reality participants were paid a fixed sum for their participation.

The majority of published studies investigate offers from 10–50% of the total stake; we also include “hyper-fair” offers of 80 and 90%. All offers were out of a total stake of £20. The number of each offer level was: 8 × 10%, 8 × 20%, 4 × 30%, 4 × 40%, 8 × 50%, 8 × 80%, 8 × 90%. The same offers were presented in each offer origin condition; thus participants received a total of 96 offers.

For repeated-measures designs, test-retest reliability is an important consideration. We tested the reliability of this version of the UG in an independent sample of healthy volunteers (N = 15; unpublished) prior to data collection. Using the rptR package^34^ to examine repeatability, we found that rejection rates in both conditions were highly reliable (FP: *R* = 0.82; random-generated: *R* = 0.69; a value of 1 represents zero within-participant variance). These estimates represent the proportion of total variance explained by between-subject variance, much like the intraclass correlation coefficient, only for binomial data.

#### Statistical analysis

The data collected was in the form of categorical (accept or reject) decisions on monetary offers. As such, the current data have been analysed using repeated-measures logistic regression, implemented with generalized estimating equations (GEE) using IBM SPSS Statistics for Windows 21 (IBM Corp., 2012), with the planned contrasts between psilocybin and the drug-free condition presented. This takes into account the correlation of responses within subjects, and produces a chi-squared statistic (χ^2^), an odds ratio (OR) and its 95% confidence interval (CI), and a p-value. The odds ratio represents the change in probability of an event (in this case, a rejection) occurring with a change in condition (fairness, offer origin).

For the sake of analysis, the offers were grouped together such that 10–20% offers were classed as ‘unfair’, 50% offers were ‘fair’, and 80–90% offers were ‘hyper-fair’. 30% and 40% offers were included in the study design to improve believability that the offers were from real people. We made the decision not include these offer levels in the analysis prior to data collection (hence the lower number of these offers presented); this decision was based on the finding that in the wider UG literature, these offer levels show wide variation in response^17^.

All reported *p*-values are Bonferroni-corrected for multiple comparisons.

### Results

With the exception of one participant in the psilocybin session, all fair and hyper-fair offers were accepted. This one participant rejected 50% and 43.8% of first-person hyper-fair offers and random-generated hyper-fair offers respectively in the psilocybin session. We will now only consider responses to unfair offers. One participant did not complete the task on the drug-free session, so these analyses are based on 19 participants.

**Figure 1:**
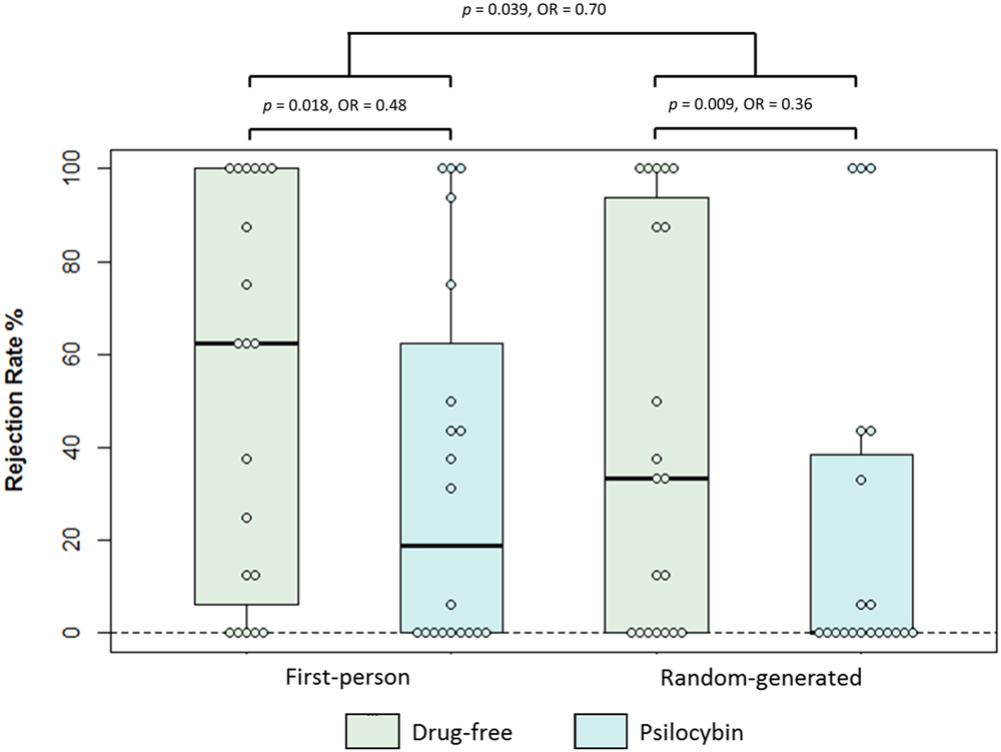
Boxplot displaying rejection rates of unfair offers in the Ultimatum Game across conditions in the psilocybin study. OR: odds ratio. Note that while the figure displays rejection rates, the statistics are based on GEE analysis which incorporates trial-by-trial data.

Figure 1 displays a boxplot of rejection rates of unfair offers across sessions. As hypothesised, there was a main effect of offer origin, such that, compared with the first-person condition, there was a reduced probability of rejecting unfair offers in the random-generated condition (χ^2^_(1,18)_ = 5.30, *OR* = 0.70, *p* = 0.039. Compared with the drug-free session, there was a reduced probability of rejecting first-person unfair offers in the psilocybin session (χ^2^_(1,18)_ = 5.53, *OR* = 0.48, *p* = 0.018, as well as random-generated unfair offers (χ^2^_(1,18)_ = 8.63, *OR* = 0.30, *p* = 0.009).

## Study two – the effects of MDMA

The data presented in this section were collected as part of a neuroimaging study investigating the effects of MDMA on social decision-making. Here, we present the behavioural results of the UG, with the neuroimaging data presented elsewhere.

### Methods

#### Participants

Twenty-one male participants were recruited from the community, all of whom had previous experience of MDMA. All participants gave written informed consent to take part in the study and were financially compensated for their time. The study received ethical approval from King’s College London’s Psychiatry, Nursing and Midwifery Research Ethics Committee (PNM/14/15-32). All experiments were performed in accordance with relevant guidelines and regulations. See the Supplementary Information S3 for exclusion criteria of this study and details of excluded participants. 20 participants completed the study (mean age 24.8 y, SD = 3.7, range = 21–37).

#### Experimental procedures

This study followed a double-blind, placebo-controlled, cross-over, counter-balanced design. Following a successful screening, participants attended two experimental study days at least one week apart (mean 9.3, SD 5.7, range 7–31).

For a full description of the experimental day, see the Supplementary Information S4. 100 mg MDMA or placebo was administered at 10:15, and the UG administered 95 minutes post-dose.

#### The Ultimatum Game

This version of the UG had three ‘offer origin’ conditions: first-person, third-party, and random computer-generated offer (random-generated).

In this version of the task, participants were led to believe that they were logged onto an online network set up as part of a collaboration between King’s College London, University College London and Imperial College London. They were told that the people they were to interact with were also logged on to the network at one of these sites. They were told that all participants would be financially reimbursed based on their responses in the UG.

This task took a total of 23.5 minutes. To protect against task fatigue, it was split into two runs, each lasting 11.75 minutes. On each run participants responded to 72 offers and were asked to make 5 offers themselves.

There is some evidence that suggests UG behaviour varies as function of total stake^35–37^. In order to asses if there was an effect of stake size we altered the stakes as well the proportion of the offers. The values were chosen to be in line with Crockett el al (2013). Including different stake values, in each run, there were eight unfair (10–20%), eight fair (45–50%) and eight hyper-fair (80–90%) offers in each condition (first-person, third-party, random-generated). See the Supplementary Information S5. By altering this aspect of the task, it was possible to analyse whether MDMA had a differential effect on high and low utility offers.

As with the version of the task used in the psilocybin study, we carried out a test-retest reliability study of this version in an independent sample of healthy participants (N = 15; unpublished data) and used the rptR package^34^ to estimate repeatability. We found that rejection rates were very reliable across sessions in each condition (FP: *R* = 0.96; TP: *R* = 0.96; random-generated: *R* = 0.84; a value of 1 represents zero within-participant variance).

#### Reward sensitivity task

In order to test if MDMA was altering participant’s sensitivity to reward, they completed a modified reaction time (RT) task. Participants saw four circles on- screen, in the layout of the arrow buttons on a standard keyboard, within a rectangular box. On each trial, participants were cued to press an arrow button as quickly and accurately as possible, by highlighting a circle on the screen. Trials were presented in four blocks, with the second and fourth rewarded. During the rewarded blocks, if participants responded faster than their average RT from the previous block, three times in a row, a pound coin appeared on the screen to indicate they would be rewarded for their performance.

Calculating the difference in average RT between rewarded and non-rewarded blocks was used as a measure of reward sensitivity.

#### Questionnaires

We asked participants to complete five subjective rating questionnaires at the end of the experimental session. Two are relevant for this manuscript.

The Social Value Orientation questionnaire^38^ is a nine-item questionnaire which requires respondents to state preferences of resource distribution, and is a validated measure of prosociality with good test-retest reliability^39,40^. The Social Reward Questionnaire^41^ is a 23-item rating scale which maps onto six factors of social reward: admiration, negative social potency, passivity, prosocial interactions, sexual relationships, and sociability.

#### Statistical analyses

Two outcome measures were collected from the UG. The first were categorical (accept or reject) responses to monetary offers. The second were continuous data of monetary offers from the participants to other players.

As with Study One, the categorical data were analysed using repeated-measures logistic regression, implemented with generalized estimating equations (GEE). Responses were grouped together for unfair (10–20% of the total stake), fair (45–50%) and hyper-fair (80–90%) offers.

As there were two runs of this task, we tested for differences across runs. Furthermore, this task was designed to test for differential effects of high/low utility. Utility was defined as per Crockett *et al*.^21^, with high utility considered more than half the maximum offer available over the course of the task. To this end, a model was first defined testing for the main effects of utility and run. Two two-way interactions of utility*treatment and run*treatment were included in this model. Finally, a model was defined to test for the main effects of treatment, offer origin (first-person, third-party, random-generated), and fairness level (unfair, fair, hyper) and their three-way interaction.

Each offer made by the participant was converted to a percentage of the total stake, and the average taken for each session. A paired-sample t-test was then performed to examine the difference in offer amount across treatment sessions.

The outcome measure of the reward sensitivity task was reaction time in milliseconds. These were analysed using a mixed effects ANOVA, with session and trial type (rewarded/unrewarded) as within-participant factors, and drug order as between subject factors.

All reported *p*-values are Bonferroni-corrected for multiple comparisons.

### Results

#### The Ultimatum Game

There was no effect of run (χ^2^_(1,18)_ = 1.00, OR = 0.87, 95%CIs 0.67–1.14, *p* = 0.319; χ^2^_(1,18)_ = 3.05) or utility (OR = 0.86, 95%CIs 0.73–1.02, *p* = 0.081). Importantly, neither run nor utility showed a statistically significant interaction with treatment (*p*s > 0.25). Based on these analyses all subsequent analyses combine runs and include all stake sizes.

**Figure 2:**
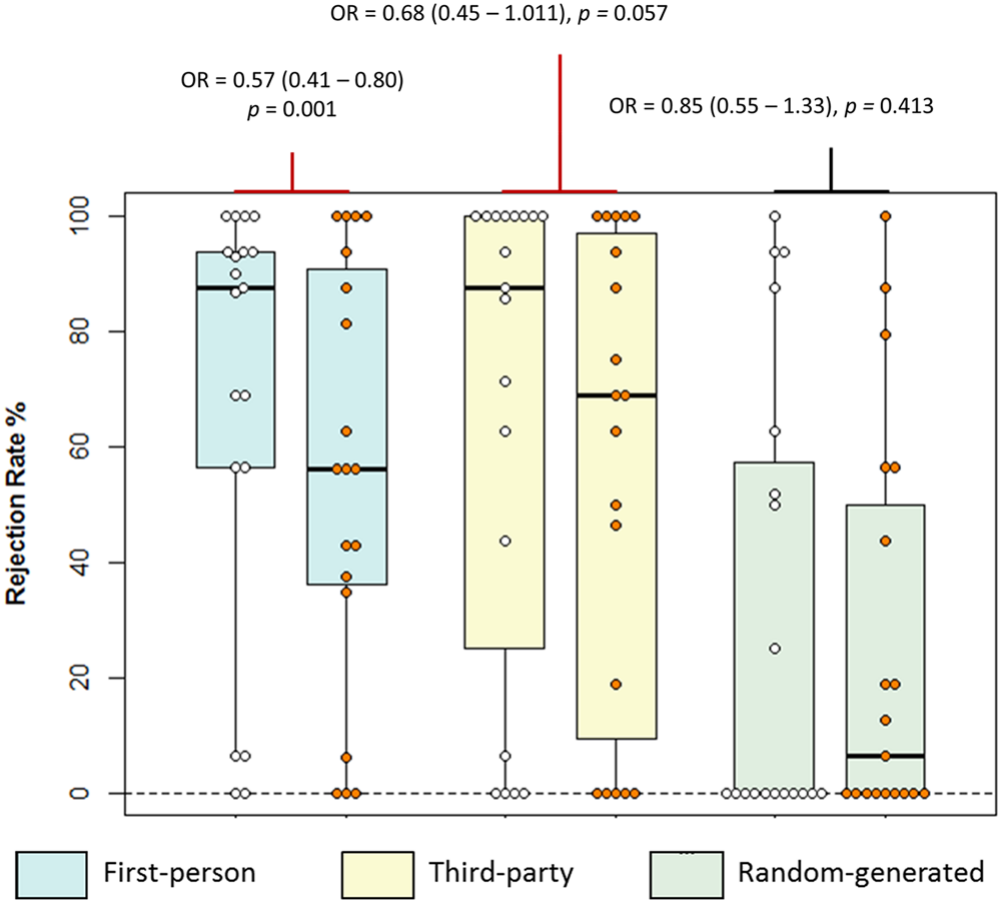
Boxplot displaying rejection rates for unfair offers in the MDMA study. White dots represent the placebo session, orange dots the MDMA session. Note that while the figure displays rejection rates, the statistics are based on GEE analysis which incorporates trial-by-trial data.

Figure 2 displays the rejection rates of unfair offers across conditions and treatment sessions.

There was no main effect of treatment (*p* = 0.888), but a main effect of offer origin, fairness, and a three-way interaction (all *p*s < 0.009). The parameter estimates of post-hoc tests revealed that MDMA produced a lower probability of rejecting unfair offers in the first-person condition (χ^2^_(1,18)_ = 11.02, OR = 0.57, 95% CIs 0.41–0.80, *p* = 0.001) but not the third-party condition (OR = 0.68, 95%CIs 0.45–1.01, *p* = 0.057). No effect of treatment was seen for unfair offers from the game-server (OR = 0.85, 95%CIs 0.56–1.30, *p* = 0.413), supported by a significant interaction. There were no other statistically significant treatment effects on rejection behaviour. We tested the effect of order by including a further factor in the model, and found no significant effect of treatment order (*p* = 0.192), nor an order-by-treatment interaction (*p* = 0.597).

We next explored the relationship between the change in first-person and third-party rejection rates. For each participant the percentage of unfair offers rejected in each of these conditions was calculated and their change across experimental sessions. There was a significant relationship between these measures (beta = 0.670, R^2^ = 0.449, *p* = 0.002).

As hypothesised, a paired t-test revealed an increase in average percentage offer from the participants during the MDMA session compared to the placebo session (placebo mean offer = 48.2%; MDMA mean offer = 55.7%; mean difference = 7.5, SD = 10.25, *t*_(18)_ = 3.17, *p* = 0.006, Cohen’s *d* = 0.82, corrected for the within-subjects correlation of r = 0.79, Morris and DeShon^42^).

#### Reward sensitivity task

A mixed-measures ANOVA showed that there was a main effect of trial type such that in both experimental sessions participants’ responded faster for the rewarded trials compared to the unrewarded trials (mean diff = −77.52 ms, *F*_(1,18)_ = 7.62, *p* = 0.014, ή^2^ = 0.30). There was no main effect of treatment session, nor a session by trial type interaction (respectively: mean diff = −4.93, *F*_(1,18)_ = 0.305, *p* = 0.588, ή^2^ = 0.02; *F*_(1,18)_ = 0.021, *p* = 0.887, ή^2^ < 0.01). There was no effect of treatment order (*F*_(1,18)_ = 0.006, *p* = 0.938, ή^2^ < 0.01).

#### Questionnaires

There was no evidence for MDMA altering prosocial or egoistic responses in the SVO (respectively: *t*_(19)_ = −1.38, *p* = 0.138; *t*_(19)_ = 0.99, *p* = 0.337).

A repeated-measures 6 (subscale) x 2 (treatment) ANOVA was carried out to test for the effect of MDMA on the SRQ. There was no main effect of treatment (F_(1,18)_ = 0.07, *p* = 0.795, ή^2^ = 0.004), but a significant main effect of subscale (F_(5,90)_ = 207.62, *p* < 0.001, ή^2^ = 0.920) and a treatment by subscale interaction (F_(5,90)_ = 2.35, *p* = 0.047, ή^2^ = 0.116). Post-hoc tests indicated that only the prosocial interactions subscale showed a change with treatment, with an increased score following MDMA (*t*_(19)_ = −3.19, *p* = 0.03).

Given this change in the SRQ’s prosociality measure, we next carried out a post-hoc regression analysis to examine if the variance of the change in this measure was related to the change in rejection rates in the UG. There was a significant relationship between the change in SRQ prosociality subscale and rejection of unfair first-person UG offers (beta = −0.498, R^2^ = 0.248, *p* = 0.035).

### Data availability

Data is available upon request.

## Discussion

We present results from two psychopharmacological studies investigating behaviour in the Ultimatum Game (UG). As hypothesised, both psilocybin and MDMA reduced rejection rates of unfair offers in a first-person (FP) condition, and MDMA increased the percentage offered when participants acted as the proposer. MDMA did not significantly reduce rejection rates in a third-party (TP) condition. An exploratory analysis found a relationship between MDMA-induced changes in prosociality, as measured by the SRQ, and change in rejection rates in the FP condition; such that those participants with greater increases in prosociality showed a greater reduction in rejection rates.

With psilocybin having potent serotonergic mechanisms^43^, these data support and extend those of Crockett *et al*.^21–23^, who reported serotonergic effects on the rejection of 30% offers in the UG. While the pharmacological profile of MDMA is broader, its largest effects are serotonergic^29,32^. Together, these results provide support for the interpretation that serotonin partially modulates UG rejection behaviour, notwithstanding other potential pharmacological mechanisms with MDMA (discussed further below).

Rejection behaviour is often considered altruistic punishment – the costly punishment of social norm violations^20,44^. Du and Chang^18^ conceptualise this as having three underlying cognitive processes: inequality aversion, cost-benefit calculation, and social reference frame. In discussing the cost-benefit calculation, the authors introduce the idea of ‘social reward currency’, such that people pay a cost for the social reward of punishing norm violations^44^. The question thus arises: do psilocybin and MDMA alter the exchange rate of this currency, such that the reward of punishment no longer outweighs the cost incurred?

To address this, one must first consider whether people are less willing to pay the cost of punishment. One approach is to consider if there is an increase in loss aversion. An effect on the cost of punishment is not supported by the literature, nor our data from the reward sensitivity task. Our task suggests that participants were neither more nor less sensitive to reward on MDMA compared to placebo, although this is not a direct test of loss aversion. Crockett *et al*.^45^ included a loss aversion parameter in a computational model of ‘moral’ decision-making, finding that increasing serotonin availability with SSRIs did not alter this. Murphy *et al*.^46^ found *reduced* loss aversion with tryptophan supplements during a decision-making paradigm.

Next, one must consider whether the reward of punishment was altered by MDMA or psilocybin, such that the supposedly ‘prosocial’ act of punishing norm violations was less rewarding. The questionnaire data in the MDMA study found an increase in the prosocial subscale of the SRQ, which was associated with the magnitude of the decrease in rejection rates. This subscale has the following items:

(i) I enjoy treating others fairly;
(ii) I enjoy feeling emotionally connected to others;
(iii) I enjoy keeping promises I make to others;
(iv) I enjoy it if someone accepts me as I am;
(v) I enjoy making someone feel happy.

Given that these items concern direct relationships with others, we argue that in the case of MDMA administration, the ‘exchange rate’ of the social reward currency was in fact shifted in favour of social reward. In Du and Chang’s^18^ conceptualisation this refers to the traditionally ‘prosocial’ considerations of punishing violations of fairness norms. Here however, it appears that concern for the direct relationship with other players was more motivating with MDMA compared to placebo. Reduced punishment behaviour has previously been interpreted in the context of harm aversion^21,22,47^, which is not inconsistent with the argument presented here – a harm aversion model could be explained by greater concern for the other players’ outcome.

Du and Chang^18^ also discuss the ‘social reference frame’, arguing for different brain regions being involved in first-person versus third-party representations. We manipulated the reference frame by having participants make decisions in a third-party condition. We predicted that there would be no change in rejection behaviour in this condition, based on the hypothesis that when not directly involved in the outcome of the interaction, fairness considerations would remain the driving force. While this was the case, it should be noted that the confidence interval around the effect size only narrowly incorporates the null effect. As such, a replication with a larger sample size is key to clarifying this result. Taken at face value, this pattern of results supports our hypothesis that MDMA increases one’s consideration of the other partner’s outcome – however, in the third-party condition, traditional ‘fairness’ considerations may take precedence due to there being multiple others to consider.

As acknowledged in our introduction, there is some debate around the psychological mechanisms underlying costly punishment in the UG. Crockett *et al*.^48^ provide data that people punish even when they know there is no possibility of the other player learning of the punishment, arguing that the motivating factor is vengeance rather than prosocial norm enforcement. However, participants in that same study rated their own motivations as being due to deterrence significantly more than retribution. As such we argue that this debate is still open. Furthermore, we argue that there likely exists a spectrum of motivating factors underlying costly punishment in the UG.

The game theoretic, ‘economically rational’ response to any offer in the UG is to accept it. Therefore, it could be argued that the effect of these compounds is to increase ‘rationality’. However, this interpretation is at odds with the finding that MDMA increased the amount offered when participants acted as proposers. Economic rationality suggests that one would offer the lowest amount possible. As such, we believe that the effect of MDMA on offers further supports the interpretation that these compounds alter social reward processing in the UG.

Recent evidence suggests that psilocybin and MDMA may be efficacious in psychiatric treatment^5,6^. The antidepressant effect of psilocybin makes its selection for study relevant, given the evidence for alterations in UG behaviour in depression^4,49^. Furthermore, the well-documented social effects of MDMA make it relevant to studying the processes reported here^50,51^.

It is significant that both sets of data reported here found the same effect on rejection in the UG. MDMA pharmacology is complex. It acts to increase the availability of serotonin through the reversal of the 5-HT transporters, as well as possibly acting as a weak direct agonist at the 5-HT2A/C receptor. Additionally, it increases the availability of dopamine and noradrenaline^29,32^. Psilocybin, on the other hand, is pharmacologically more selective; a mixed serotonergic agonist, with strong evidence that its ‘psychedelic’ effects result from 5-HT2A receptor agonism^52^. This supports an interpretation that the behavioural effects seen here are a result of serotonergic modulation.

Although the 5-HT effect is far greater^53^, the role of dopamine and noradrenaline in the MDMA response should not be discounted, particularly since there is limited evidence for indirect dopaminergic modulation via psilocybin’s 5-HT activity^54^.

The use of specific receptor antagonists alone and as part of blocking studies would be an important step to further characterise the receptor mechanisms underlying the behavioural changes reported above. Equally important, however, the data reported here contribute to our understanding of the cognitive and subjective effects of these compounds, regardless of the specific pharmacological mechanisms underlying them.

A major limitation of the current presentation is the open-label nature of the psilocybin study, and its lack of counter-balancing. However, the tasks used in both studies had excellent test-retest reliability, thus allowing confidence in the findings presented. Furthermore, while the MDMA study was placebo-controlled, there is recognition in the field of psychedelic/psychotomimetic research that inert placebos do not sufficiently blind participants to the treatment they receive. There is currently no recognised standard choice for an active placebo for either of the compounds studied here.

Investigation of the dose-response relationship would be beneficial to both studies reported in this manuscript. In the absence of true blinding to the active compound, assessment of the dose-response on the performance on cognitive tasks would extend the interpretability of specific drug effects and allow a detailed assessment of the relationship between the subjective and cognitive effects.

These studies used different versions of the UG, in different settings – one inside the scanner, the other outside. Validation studies in our group (data not shown) show that both versions produce very similar rejection rates and show good test-retest reliability. Rejection rates for the MDMA version were similar in the placebo condition (in the scanner) compared to when the task was validated (outside of the scanner).

It should also be noted that the studies reported here recruited only male participants. Psychopharmacological studies are particularly sensitive to differences in hormonal levels across participants. As such, single-sex studies have more power to detect a given effect. However, it is important to note that previous studies have found gender differences in the UG^55,56^. While the psilocybin response does not appear to be moderated by gender^57^, gender differences in the subjective effects of MDMA *have* been reported^58^. This raises the intriguing question of whether the changes in social reward processing posited in the current manuscript is generalizable across genders.

Related to the above, all participants in the reported studies had prior drug experience relevant to the drug they were administered in the study, and had reported only positive previous experiences. These selection criteria were an ethical requirement. However, such criteria have the potential to bias the participants, thus affecting the generalisability of the findings.

The research presented here characterises the effects of psilocybin and MDMA on social decision-making, and provides further support for the role of serotonergic modulation on these processes. This work suggests that underlying responses to interactive social situations are complex mechanisms that are at least partially driven by serotonin receptor activity; something that can be further investigated with selective receptor blockade studies. Social decision-making is a burgeoning field increasingly being investigated in relation to psychiatry. Doing so can help to clarify how higher-level cognitive processes are disrupted in psychiatric disorders. We argue that MDMA and psilocybin reduce costly punishment through alteration of the ‘social reward currency’, by increasing one’s concern for the outcome of interacting partners.

## Electronic supplementary material

Supplementary materials
