# Supplementary material for: Psilocybin and MDMA reduce costly punishment in the Ultimatum Game
Source: Sci Rep. 2018 May 29;8:8236. doi: 10.1038/s41598-018-26656-2 (PMC5974271; doi:10.1038/s41598-018-26656-2)
Supplement: Supplementary file 1 — Supplementary materials [file 41598_2018_26656_MOESM1_ESM.pdf]

Title: Psilocybin and MDMA reduce costly punishment in the Ultimatum Game

Authors: Anthony S Gabay[1],Robin Carhart-Harris[3], Ndaba Mazibuko[1], Matthew J Kempton[1,2] , Paul D Morrison[2], David Nutt[3], Mitul A Mehta[1]

[1] Department of Neuroimaging, Institute of Psychiatry, Psychology & Neuroscience, King's College London

[2] Department of Psychosis Studies, Institute of Psychiatry, Psychology & Neuroscience, King's College London

[3] Psychedelic Research Group. Neuropsychopharmacology Unit. Centre for Academic Psychiatry, Imperial College London.

Supplementary materials

S1: Exclusion criteria for Study One and reasons for participants excluded

Exclusion criteria included: personal history of psychiatric illness (assessed with the Mini-International Neuropsychiatric Interview, Sheehan et al., 1998); first-order relative with a history of psychotic illness; evidence of cardiac (assessed with ECG), hepatic, renal, gastrointestinal (assessed with standard blood screening) or neurological disorders; excessive use of caffeine (> six cups of coffee per day) and alcohol (> 28 units per week); current use of medication; failure of drugs of abuse test at screening or on either study day (drugs tested for: amphetamine, barbiturates, benozodiazepines, cocaine, THC, methadone, methamphetamine, opiate, phenylcyclidine, tricyclic antidepressants). Participants were only included in this study if they had at least one previous experience with a hallucinogenic drug. Participants were excluded if any previous experience could be described as 'negative', or a 'bad trip'. We did not collect data on lifetime use.

Three participants did not complete the study: the QTc reading of one participant's ECG exceeded the upper limit specified in the study protocol on the day of testing; one participant experienced high anxiety prior to the psilocybin dosing on his first session and withdrew from the study; one participant tested positive for cocaine on the morning of his second session.

S2: Full description of the experimental study day for Study One

Participants arrived at the study centre at 08:30, at which time we repeated neurological, cardiac, and general health safety checks to ensure nothing had changed since their screening visit. At 10:00 participants were dosed with either 125mg saracatinib or placebo, orally. At 30 minutes and 120 minutes post-dose participants gave a blood sample. At 180 minutes post-dose participants completed some questionnaires and were retrained in the tasked they were to perform in the scanner. At 240 minutes post-dose participants entered the scanner. The scanning session lasted 90 minutes, with an infusion of 2mg psilocybin over 2 minutes occurring approximately 40 minutes into the scanning session (280 minutes post saracatinib).

Following the scanning session, a further blood sample was taken and participants completed a questionnaire of subjective effects, the UG (340 minutes post saracatinib) and the Affective Bias task (355 minutes post saracatinib). Participants then completed further questionnaires before commencing discharge procedures. The study typically finished at around 17:00.

S3: Exclusion criteria for Study Two

Exclusion criteria included: personal history of psychiatric illness; first-order relative with a history of psychotic illness; evidence of cardiac, hepatic, renal, gastrointestinal or neurological disorders; excessive use of caffeine and alcohol; current use of medication; failure of drugs of abuse test at screening or on either study day. Only participants with previous experience of MDMA were included in this study. Participants were only included in this study if they had at least one previous experience with MDMA. They were also required to have not used MDMA in the three months leading up to their involvement in the study. We did not collect data on lifetime use.

One participant withdrew from the study after his first visit. This was unrelated to his participation, and unblinding revealed that he received placebo.

S4: Full description of the experimental study day for Study Two

Participants arrived at the study centre at 08:45, at which time we repeated physical health screening checks to confirm they were still eligible to take part. At 10:00 a pre-dose blood sample was taken to assess baseline plasma oxytocin levels. At 10:15

participants were dosed with either 100mg MDMA or placebo, orally. At 45 minutes post-dose participants gave another blood sample to assess plasma oxytocin and MDMA levels. Between these samples participants were retrained in the tasks. At 75 minutes post-dose participants entered the scanner. The scanning session lasted 90 minutes, with the first task beginning approximately 20 minutes into the session. Prior to the fMRI tasks we collected structural scans, resting state data and arterial spinal labelling data. These data are not presented in this manuscript.

The timing for the MRI session was chosen because the Tmax of MDMA ranges between 1.5 – 3 hours (Kolbrich et al., 2008), and subjective effects peak and remain stable between 1 and 3 hours (Harris et al., 2002), meaning functional acquisitions would fall within these time points.

S5: Full list of offers in the Ultimatum Game used in Study Two

Table S 1: Table B-1: Full list of UG offers presented in the MDMA study. Ordered by condition and then percentage of the total stake. Shading reflects unfair (10-20%), fair (45-50%), and hyper-fair (80-90%) offers. FP: first person; TP: third-party; GS: game server

Offer from…	% of total stake	Total stake	Utility
Run one			
FP	10	30	Low
FP	10.94	32	Low
FP	11.96	46	High
FP	13.04	11.5	Low
FP	13.05	38.3	High
FP	17.11	38	High
FP	20	5	Low
FP	20	37.5	High
FP	46.15	6.5	Low
FP	46.67	7.5	Low
FP	47.06	8.5	Low
FP	47.62	10.5	High
FP	48.15	13.5	High
FP	49.18	12.2	High
FP	50	2	Low
FP	50	3	Low
FP	80	2.5	Low
FP	81.08	3.7	Low
FP	82.35	8.5	High
FP	84.62	6.5	High
FP	86.36	11	High
FP	86.67	7.5	High

FP	90	5	Low
FP	90.91	1.1	Low
TP	11.97	58.5	High
TP	13.05	38.3	High
TP	13.16	19	Low
TP	14.04	28.5	Low
TP	17.91	33.5	High
TP	19.15	23.5	Low
TP	20	37.5	High
TP	20	5	Low
TP	46.67	7.5	Low
TP	47.37	9.5	Low
TP	47.62	10.5	High
TP	48.15	13.5	High
TP	50	19	High
TP	50	15	High
TP	50	3	Low
TP	50	2	Low
TP	80	5	Low
TP	82.35	8.5	High
TP	83.33	3	Low
TP	83.33	6	High
TP	85.71	7	High
TP	88.24	1.7	Low
TP	88.89	9	High
TP	90.91	1.1	Low
GS	10	30	Low
GS	11.97	58.5	High
GS	13.04	11.5	Low
GS	13.05	38.3	High
GS	14.04	28.5	Low
GS	17.02	47	High
GS	17.91	33.5	High
GS	20	5	Low
GS	46.67	7.5	Low
GS	47.06	8.5	Low
GS	47.62	10.5	High
GS	49.02	5.1	Low
GS	49.18	12.2	High
GS	50	16	High
GS	50	19	High
GS	50	2	Low
GS	80	5	Low
GS	80	2.5	Low

GS	83.33	6	High
GS	84.62	6.5	High
GS	88.24	8.5	High
GS	88.89	9	High
GS	90	5	Low
GS	90.91	1.1	Low
Run two			
FP	11.97	58.5	High
FP	13.16	19	Low
FP	14.04	28.5	Low
FP	17.02	47	High
FP	17.91	33.5	High
FP	17.92	53	High
FP	18.35	10.9	Low
FP	19.15	23.5	Low
FP	44.44	4.5	Low
FP	47.37	9.5	Low
FP	47.83	11.5	High
FP	48.28	14.5	High
FP	49.02	5.1	Low
FP	50	19	High
FP	50	16	High
FP	50	15	High
FP	80	5	Low
FP	83.33	6	High
FP	83.33	3	Low
FP	85.37	4.1	Low
FP	85.71	7	High
FP	88.24	8.5	High
FP	88.24	1.7	Low
FP	88.89	9	High
TP	10	30	Low
TP	10.94	32	Low
TP	11.96	46	High
TP	13.04	11.5	Low
TP	17.02	47	High
TP	17.11	38	High
TP	17.92	53	High
TP	18.35	10.9	Low
TP	44.44	4.5	Low
TP	46.15	6.5	Low
TP	47.06	8.5	Low
TP	47.83	11.5	High
TP	48.28	14.5	High

TP	49.02	5.1	Low
TP	49.18	12.2	High
TP	50	16	High
TP	80	2.5	Low
TP	81.08	3.7	Low
TP	84.62	6.5	High
TP	85.37	4.1	Low
TP	86.36	11	High
TP	86.67	7.5	High
TP	88.24	8.5	High
TP	90	5	Low
GS	10.94	32	Low
GS	11.96	46	High
GS	13.16	19	Low
GS	17.11	38	High
GS	17.92	53	High
GS	18.35	10.9	Low
GS	19.15	23.5	Low
GS	20	37.5	High
GS	44.44	4.5	Low
GS	46.15	6.5	Low
GS	47.37	9.5	Low
GS	47.83	11.5	High
GS	48.15	13.5	High
GS	48.28	14.5	High
GS	50	3	Low
GS	50	15	High
GS	81.08	3.7	Low
GS	82.35	8.5	High
GS	83.33	3	Low
GS	85.37	4.1	Low
GS	85.71	7	High
GS	86.36	11	High
GS	86.67	7.5	High
GS	88.24	1.7	Low
